# Detection of Antibody-Dependent Cell-Mediated Cytotoxicity—Supporting Antibodies by NK-92-CD16A Cell Externalization of CD107a: Recognition of Antibody Afucosylation and Assay Optimization

**DOI:** 10.3390/antib12030044

**Published:** 2023-06-27

**Authors:** Judith Cruz Amaya, Bruce Walcheck, Julie Smith-Gagen, Vincent C. Lombardi, Dorothy Hudig

**Affiliations:** 1 Department of Microbiology and Immunology, School of Medicine, University of Nevada, 1664 N. Virginia St., Reno, NV 89557, USA; 2 Department of Veterinary and Biological Sciences, Center for Immunology and Masonic Cancer Center, University of Minnesota, 295J AS/VM Building, 1988 Fitch Avenue, Saint Paul, MN 55108, USA; 3 School of Community Health Sciences, University of Nevada, 1664 N. Virginia St., Reno, NV 89557, USA

**Keywords:** antibody-dependent cell-mediated cytotoxicity, ADCC, NK-92 cells, CD16A, CD107a, fucosylation, GA101

## Abstract

Antibody-dependent cell-mediated cytotoxicity (ADCC) by natural killer (NK) lymphocytes eliminates cells infected with viruses. Anti-viral ADCC requires three components: (1) antibody; (2) effector lymphocytes with the Fc-IgG receptor CD16A; and (3) viral proteins in infected cell membranes. Fc-afucosylated antibodies bind with greater affinity to CD16A than fucosylated antibodies; individuals’ variation in afucosylation contributes to differences in ADCC. Current assays for afucosylated antibodies involve expensive methods. We report an improved bioassay for antibodies that supports ADCC, which encompasses afucosylation. This assay utilizes the externalization of CD107a by NK-92-CD16A cells after antibody recognition. We used anti-CD20 monoclonal antibodies, GA101 WT or glycoengineered (GE), 10% or ~50% afucosylated, and CD20-positive Raji target cells. CD107a increased detection 7-fold compared to flow cytometry to detect Raji-bound antibodies. WT and GE antibody effective concentrations (EC_50_s) for CD107a externalization differed by 20-fold, with afucosylated GA101-GE more detectable. The EC_50_s for CD107a externalization vs. ^51^Cr cell death were similar for NK-92-CD16A and blood NK cells. Notably, the % CD107a-positive cells were *negatively* correlated with dead Raji cells and were nearly undetectable at high NK:Raji ratios required for cytotoxicity. This bioassay is very sensitive and adaptable to assess anti-viral antibodies but unsuitable as a surrogate assay to monitor cell death after ADCC.

## 1. Introduction

Antibody-dependent cell-mediated cytotoxicity (ADCC) by natural killer (NK) lymphocytes is a form of long-lasting immunity that provides protection against many viruses (reviewed [1]). Antibodies that support ADCC (IgG1 and IgG3 [2,3]) are sustained throughout an individual’s lifetime. IgG1 antibodies will support killing at concentrations as low as 0.1 ng/mL [4], indicating that ADCC will persist even as antibody levels decrease with time. Effective antibodies include those that are unable to neutralize viral infectivity [5]. These antibodies recognize many viral epitopes in addition to those of viral receptor-binding domains (RBDs) that are essential for viral entry into cells [6]. The NK cells responsible for ADCC are abundant, circulate throughout the body, and constitute 10–20% of all the blood lymphocytes. CD16A expressed by NK cells will recognize the Fc region of IgG antibodies attached to viral proteins in the plasma membranes of infected cells. ADCC by NK cells kills immediately, unlike cytotoxic memory T cells that need a recall response before they can effectively kill [7,8]. In light of the potency of ADCC, quantification of ADCC-supportive antibodies is desirable to assess the strength of long-term anti-viral protection.

Straightforward quantification of antibodies is unable to predict the ability of antibodies to support ADCC because of post-translational modifications of the Fc [9,10,11,12]. In particular, fucosylation of the antibody Fc reduces its affinity for CD16A [13,14] and thereby reduces the ability of fucosylated antibodies to support ADCC [4,15,16]. Intra-donor variations in afucosylation range between 2% and over 24% of IgG1 molecules, e.g., for antibodies to SARS-CoV-2 [17,18]. Currently, measurements of afucosylation for antigen-specific antibodies utilize affinity purification of the antibodies and mass-spectrometry; however, these techniques have limited clinical use because they are costly in terms of both money and time. This approach is also impractical due to the limited volumes of clinical serum samples. Simpler and faster assays are needed to titrate the effective antibodies in serum samples. Here, we report a fast NK cell-based assay for the detection of specific antibodies that support ADCC, which is independent of antibody purification and requires only small amounts of antibodies.

The assay depends on NK cell externalization of lysosomal-associated membrane protein 1 (LAMP-1), also designated as CD107a [19,20,21]. CD107a lines the inner membranes of lysosomes and intracellular granules. In NK and CD8 cytotoxic T cells, the granules contain cytotoxic proteins (perforin and granzymes) that are released during killing (reviewed [22]). During cytotoxic granule release, the granule membrane fuses with and is incorporated into the cytotoxic cell’s extracellular plasma membrane. In the process, CD107a becomes externalized [23]. Externalization of CD107a is a hallmark of receptor engagement of ‘effector’ killer cells with ‘target’ cells for both cytotoxic T cells [24,25] and NK cells [25]. CD107a has been used as a surrogate marker to indicate cytotoxic activity. A previous report of CD107a externalization by NK-92-CD16A cells monitored the development of antibodies towards influenza after vaccination or natural infection [26]. This assay with microtiter-plated antigens required hours for NK cell externalization and tittered rather than quantified the influenza-specific antibodies. Also, as we report here, CD107a externalization has limitations as a measurement of NK cellular activity. At least in the case of ADCC, CD107a is best viewed as an indicator of antibody-CD16A receptor engagement rather than as a surrogate marker for ‘target’ cell death.

The sensitivity of CD107a externalization provided the basis for our design of an optimized assay to compare antibodies with post-translational modifications. Our ultimate goal is to monitor the effects of natural fucosylation of human anti-viral antibodies that support ADCC. There are three components to our assay. The first component is an invariant, renewable, and stable source of NK cells expressing CD16A. An NK-92-CD16A tumor cell line [27,28] meets this criterion by having the following properties: (a) immortality of the parental line NK-92 [29,30]; (b) excellent cytotoxic activity; and (c) the expression of CD16A and GFP as a result of lentiviral transformation. The second component is an internal antibody standard for calibration to control for intra-experimental variations. The standard for reference is the monoclonal antibody (mAb) GA101-WT anti-CD20 that is ~10% afucosylated, comparable to human afucosylation of total circulating IgG1 [31,32]. The third component is ‘target’ cells with antigen expression. Here, we used the Raji B cell tumor [33] that expresses consistent CD20 and is a poor target for NK activity in the absence of antibodies. In addition, production of the GA101 mAb in genetically modified CHO cells produces glycoengineered anti-CD20 mAb (GA101-GE) [34] with a protein identical in sequence with GA101-WT but 50% afucosylated and marketed as a therapeutic antibody Gazyva^®^. The 40–50% afucosylation is the typical upper range for naturally occurring anti-viral antibodies (30% afucosylated to the spike (S) protein of SARS-CoV-2 ([17,35]); 40% to HBV and 80% to human CMV [17]). Since our ultimate application is anti-viral antibodies, we included an evaluation of the effects of heat inactivation and formaldehyde that are used to inactivate viruses.

We describe an improved NK-92-CD16A CD107a-based biological assay for antibodies that will support ADCC. The NK cell CD107a externalization detects lower levels of antibodies bound to the target cells than is detectable by flow cytometry. To the best of our knowledge, we are the first to quantify the antibody detection and to demonstrate that this cellular CD107a externalization detects differences in antibody fucosylation. We observed unexpected conditions that influenced CD107a externalization: the lower the effector NK cell to Raji target cell ratio (E:T), the greater the CD107a expression. *Remarkably, the high E:T conditions that supported the killing of Raji cells failed to support CD107a externalization*. Overall, we report that the NK CD107a assay is (1) very effective for the measurement of antibodies that will support ADCC and (2) unsuitable as a surrogate assay to monitor target cell death by ADCC.

## 2. Materials and Methods

### 2.1. Cell Lines and Peripheral Blood Mononuclear Cells (PBMCs)

NK-92-CD16A cells were derived by the author B.W. from a cell line ATCC CRL-2407 and lentivirus-transformed to express CD16A AA158valine and green fluorescent protein [28]. Cells were cultured per original ATCC instructions with alpha Minimum Essential Media containing L-glutamine and sodium pyruvate (Gibco (Waltham, MA, USA)), 0.2 mM 2-mercaptoethanol, 0.2 mM inositol, 0.02 mM folic acid, 12.5% horse serum (Gibco), 12.5% fetal bovine serum (FBS) (Biowest, Riverside MO, USA), 1% pen-strep (Gibco), and 1000 U/mL Teceleukin recombinant interleukin 2 (Roche, Basel, Switzerland). Cells were maintained at 5% CO_2_ and 37 °C.

Raji cells (ATCC CCL-86) [33] were cultured in RPMI media with L-glutamine (GenClone, El Cajon, CA, USA), 10% FBS, and 1% pen-strep at 5% CO_2_ and 37 °C.

Both cell lines were regularly tested for mycoplasma (Lonza MycoAlert, Basel, Switzerland) and were negative.

Peripheral blood mononuclear cells (PBMCs) from the healthy donors were isolated at UNR by ficoll-hypaque density gradient centrifugation [36] as described in [37], wherein the ADCC was originally reported for these donors as data points of Figure 2B. ADCC and EC_50_ assays, FcγRIII genotyping, CD16A-positive NK cell TruCounts^®^ (Becton Dickenson no. 340334), and immunophenotyping were determined in the original study. The use of human subjects was approved by the institutional review board of the University of Nevada, Reno School of Medicine. Written informed consent was obtained from the blood donors.

### 2.2. Antibodies for CD107a Exocytosis and ADCC

Humanized anti-CD20 monoclonal antibody was used to support ADCC. There were two glycosylated forms of one monoclonal antibody. One form was highly afucosylated obinutuzumab (brand name Gazyva^®^, which is used therapeutically; also reported as glycoengineered GE GA101) [34,38,39]. This antibody was produced in CHO cells that were genetically modified to reduce fucosylation of antibodies [34]. The other WT antibody was ~10% afucosylated and produced in standard CHO cells. The extent of Fc-fucosylation for obinutuzumab/GE GA101 is ca. 50%, and for WT GA101 is greater than 90% (communicated by Christian Klein, Roche Innovation Center Zurich, Switzerland).

### 2.3. Detection of CD107a Externalization

When cytotoxic T [40] or NK [41] cells kill other cells, they release perforin and granzymes from intracellular granules. The membranes of the cytotoxic granules and the cells’ plasma membrane fuse during the release of the granule contents. Simultaneously, an inner membrane granule protein LAMP-1/CD107a is externalized and becomes part of the killer cell’s plasma membrane. Here, we detected external CD107a of un-permeabilized NK cells with PE-mouse mAb anti-CD017a.

**Figure A1:**
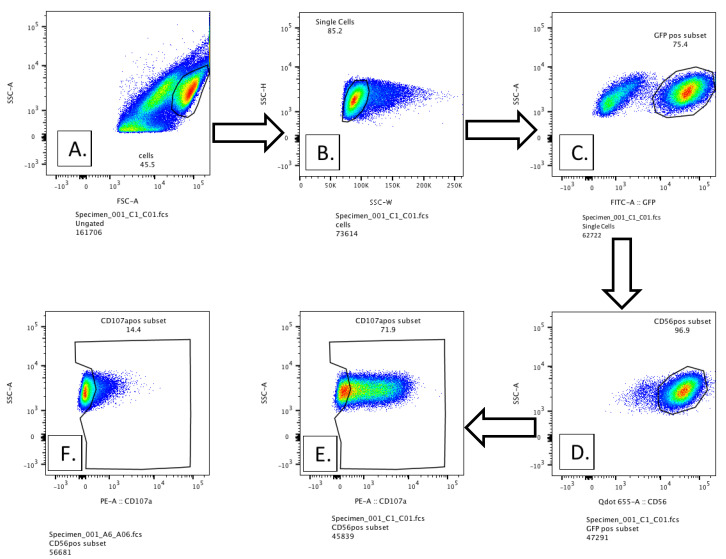
Flow cytometric gating to detect CD107a externalization. (**A**–**E**) sequential gating for CD107a-positive NK-92-CD16A cells incubated with antibody and Raji cells. The assay conditions were an NK to Raji E:T of 1:2, with or without 10 ng/mL GA101-GE antibody, and 40 min of incubation at 37 °C. (**A**) Starting NK and Raji cells; (**B**) gating for single cells; (**C**) gating for GFP-bright NK-92-CD16A cells; (**D**) gating for CD56 bright GFP positive double-positive cells; (**E**) gating for the % CD107a-positive cells. (**F**) NK cells with Rajis without mAb. The data are from experiment JCA044, illustrated in Figure 1.

Specifically, Raji “target” cells (500,000 cells at 2.0 × 10^6^/mL in 0.25 mL) were incubated for 30 min at RT with various concentrations of GA101-GE or GA101-WT antibody in duplicates. A control for exocytosis was 50 ng/mL phorbol myristic acid (PMA, Sigma, St. Louis, MO, USA) and 50 ng/mL ionomycin (StemCell, Vancouver, BC, USA). NK-92-CD16A cells to produce various effector-to-target cell ratios were added in 0.25 mL. (For example, for an E:T of 1:2, 250,000 cells at 1.0 × 10^6^/mL of NK-92-CD16Acells in 0.25 mL were added). Tubes were centrifuged at 1000 rpm for 3 min to bring the effector and target cells together and then incubated at 5% CO_2_ and 37 °C for 0, 20, 40, 60, or 120 min. After incubation, the cells were placed on ice, and ice-cold isotonic Na_2_EDTA in FACS buffer (Sheath fluid (BioSure, Grass Valley, CA, USA), 1% FCS, and 0.09% NaN_3_) was added to a final concentration of ~3 mM EDTA to chelate calcium and stop exocytosis. The tubes were centrifuged at 1200 rpm for 10 min, the supernatants were decanted, and the cells were left in minimal volumes (~50 μL) to promote good labeling with fluorescent antibodies. Cells were stained for 30 min at RT with an antibody panel containing PE-αCD107a (clone H4A3, BD Bioscience, San Jose, CA), APC-αCD19 (clone H1B19, BioLegend, San Diego, CA, USA), PacBlue-αCD45 (clone H130, BioLegend), and BV650-αCD56 (clone HCD56, BioLegend). The stained cells were washed twice with FACS-EDTA buffer and 0.5% formaldehyde fixed before the flow cytometric analysis. The instrument used was a BD Biosciences Special Order Research Product (SORP) LSR II analytical flow cytometer with a High Throughput Sampler. The data were analyzed using FlowJo version 10 (FlowJo, LLC, Ashland, OR, USA). The percentage of NK-92s cells that were CD107a-positive cells was determined by dividing the number of CD107AposCD56posGFPpos NK-92 cells by the total number of CD56posGFPpos NK-92 cells (Appendix A Figure A1E).

Antibody EC_50_. The effective concentration of antibody needed to support 50% of maximal CD107a externalization or ADCC (EC_50_ [42]) is a measurement of NK cell receptor engagement of antibodies on the target cells. For CD107a, the maximal externalization was the percent of cells with CD107a antibodies minus the CD107a externalization of NK cells without mAb. This subtraction was necessary because of variation in NK CD107a externalization without mAb. Half of this ADCC-specific externalization was added to the background NK CD107a externalization and used as the Y in the linear equation y = mx + b to solve for X the log_10_ of antibody for the EC_50_. Note, in the case of EC_50_s for ^51^Cr release, the NK cytotoxicity without mAb was negligible, so Y was half the maximal ^51^Cr release at high antibody concentrations.

### 2.4. Flow Cytometric Detection of Antibodies Bound to Raji Cells

This method was used to compare cellular CD107a with detection of Raji-bound antibodies that stimulated the CD107a externalization. Raji cells were pre-incubated with dilutions of GA101 WT or GE mAb for 30 min. After incubation, the cells were washed twice and then labeled for 30 min at RT with AF594-conjugated affiniPure donkey anti-human IgG (H + L) (Jackson ImmunoResearch, West Grove, PA, USA). Using this procedure, we also verified that the CD20 antigen-binding properties of the WT and the GE antibodies were identical.

### 2.5. Cytotoxicity Assays

Targets release internalized ^51^Cr into the supernatant when they die. Target Raji cells were labeled with Na^51^CrO_4_ (Perkin Elmer, Waltham, MA, USA) [43]. Raji and effector NK-92-CD16A cell counts were determined by Trypan blue (MilliporeSigma, Burlington, MA, USA) exclusion. Assays were in V-bottom plates (Costar 3894, 96 well) in 0.2 mL with 1 × 10^4^ Raji cells per well in quadruplicate. There were two experimental formats, one for antibody EC_50_s and another for the effects of different E:T ratios. For the antibody EC_50_ determinations, GA101 WT or GE antibodies were diluted 2-fold. Radiolabeled Raji cells (10^4^) were added to each well and incubated for 30 min at RT. NK-92-CD16A cells for a final E:T of 16:1 were added to each well. For evaluation of E:T effects, NK-92-CD16A cells were diluted 2-fold to create the E:Ts. Radiolabeled Raji cells were pre-incubated with GA101 WT or GE for 30 min at room temperature and then added to the wells with the varying NK-92-CD16A cells. Plates were centrifuged at 1000 rpm for three minutes to bring the effector and target cells together and incubated at 5% CO_2_ and 37 °C for forty min, two hours, or four hours. After incubation, ~3 mM Na_2_EDTA was added to each well, and then the plates were centrifuged at 1200 rpm for 10 min. Half of the cell-free supernatant was removed for analysis in a Perkin-Elmer Wizard gamma counter. The spontaneous release was the average leak rate of target cells without effectors; the maximum release was the radioactivity released by the target cells lysed with 1% SDS. The calculated % specific release is a measure of the dead target cells. Percent specific release (SR) was calculated using the following formula:

%SR = [(Experimental counts − Spontaneous Release)/(Max − Spontaneous Release)] × 100

### 2.6. Effects of Anti-Viral Biosafety Conditions

Heat inactivation of sera. Human male AB serum (Sigma Aldrich, Visalia, CA, USA) was heat inactivated at 56 °C for 30 min with and without addition of GA101 WT. The heated serum with GA101 was diluted with heated serum without mAb to make two mAb concentrations. The solutions were incubated with Raji cells for 30 min, then assayed with NK-92-CD16A cells for 40 min.

Formaldehyde treatment. The effects of formaldehyde could impair NK recognition of denatured anti-target antibodies and/or denature the epitope of CD107a. For effects on the bound mAb, Raji cells with antibody were washed once, then treated with 1.0% formaldehyde for 15 min, washed twice to remove formaldehyde, counted, and used as in Section 2.3 to elicit CD107a externalization. For the formaldehyde effects on CD107a, cells were treated immediately after incubation with or without 0.5% formaldehyde for 15 min and washed twice before labeling with fluorescent antibodies.

### 2.7. Graphics

Graphics were made using GraphPad Prism 9 (V 9.5.1.733 for Windows, GraphPad Software, San Diego, CA, USA) and modified using Microsoft PowerPoint V2019.

### 2.8. Statistical Analyses

CD107a assessments. The FlowJo “compare population” tool was used for Overton subtractions [44]. Excel Student’s *t*-tests [45] were applied to compare duplicate samples with other duplicate samples, using one-tailed and type 2 (two-sample equal variance [homoscedastic]) settings.

^51^Cr-cytotoxicity assays. Data were calculated with Microsoft Excel, and the significance of comparisons was assessed with paired 4-well sets using Student’s *t*-tests.

**Figure 1:**
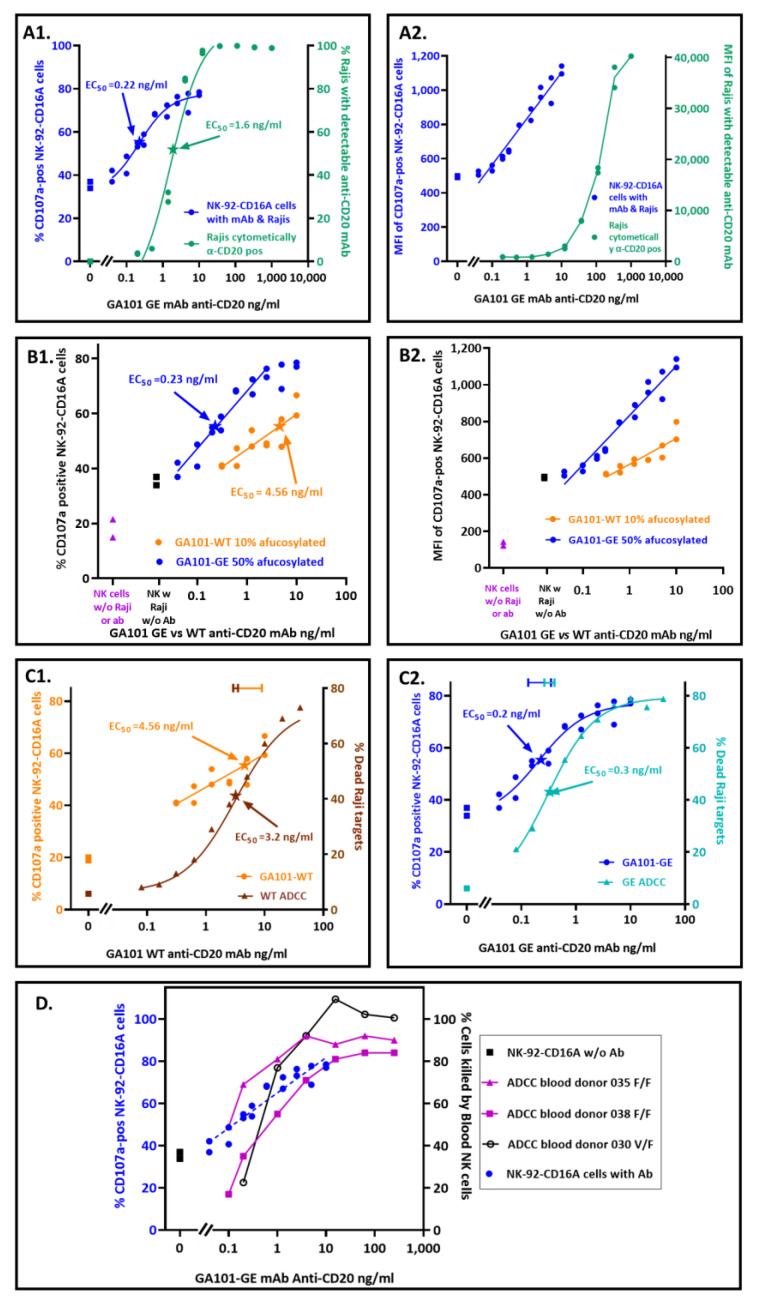
Assay by NK-92-CD16A cell externalization of CD107a for antibodies that can support ADCC. The Raji cells were pre-incubated with GA101 anti-CD20 antibodies, then NK-92-CD16A cells were added at a 1:2 effector NK to Raji target ratio (E:T), and the cells incubated for 40 min at 37 °C. NK and Raji cells were also incubated without antibodies to measure CD107a externalization associated with NK activity. (**A**) Antibody detection by NK CD107a vs. by fluorescent secondary anti-IgG to Raji-bound antibodies. For the anti-CD20 antibody bound to Raji cells, the Rajis were stained with AF647-labeled donkey anti-human IgG. Both PE-anti-CD107a and AF647 anti-human IgG were detected by flow cytometry. (**A1**) Detection of target-cell bound antibody by NK CD107a or by fluorescent anti-human IgG. The EC_50_s for each method are indicated with arrows. EC_50_ values are the effective concentrations of anti-CD20 that elicited 50% of maximum NK-92 antibody-specific (ADCC minus NK) CD107a externalization. The values for NK activity (without antibodies) are indicated by square symbols. (**A2**) The median fluorescent intensities (MFIs) of the CD107a-positive NK cells or AF647-anti-human IgG labeled Raji cells. The NK MFIs are for only the CD107a-positive cells. (**B**) CD107a externalization in response to antibodies with different Fc-fucosylation. The antibodies are from one mAb clone, GA101. The WT antibody is ~10% afucosylated; the GE antibody 50% afucosylated. (**B1**) EC_50_s for antibodies that differed in fucosylation. The EC_50_s associated with afucosylation were 20-fold apart in this experiment (*p* < 0.05); similar differences were observed for three other experiments. (**B2**) The MFIs of the CD107a positive cells. The MFIs are for the CD107a positive in (**B1**). The CD107a per cell increased with afucosylation (*p* < 0.001). (**C**) Antibody concentrations for CD107a externalization vs. death by ADCC. The CD107a values are the data of (**B1**). (**C1**,**C2**) Antibody EC_50_s for CD107a vs. for cell death, with GA101-WT or GA101-GE antibody. NK CD107a was determined at an E:T of 1:2. ADCC-mediated death was determined with ^51^Cr-Raji cells at an E:T of 20:1. Both assays were stopped at 40 min. The 95% confidence limits for each EC_50_ are color-coded and indicated at the top of the graphs. (**D**) Antibody detection by NK-92CD16A cell CD107a or by ADCC by peripheral blood NK cells. The donors’ genotypes encoding CD16A AA158, either lower affinity for Fc-IgG phenylalanine (F) or higher affinity valine (V), are indicated. The CD16A-positive blood NK cell to target (E:T) ratios were 4:1, 3:1, and 1:1 for donors 030, 035, and 038, respectively.

Comparisons of EC_50_s. For comparison of the linear regressions in Figure 1(B1,B2), the data were evaluated using analysis of variance with SPSS Statistics (IBM, version 28, Armonk, NY, USA). For comparison of the EC50s for CD107a vs. 51Cr cytotoxicity in Figure 1(C1,C2), the 95% confidence intervals were calculated for each EC_50,_ and then the confidence intervals were compared for overlap.

## 3. Results

### 3.1. Rationale

Our rationale was to use NK-92-CD16A cells as biosensors for antibodies that bind to CD16A, the receptor for NK cell-mediated ADCC. The use of cells was designed to (a) improve CD16A recognition of afucosylated antibodies, (b) exploit the avidity of multiple NK FcR-target antibody Fc interactions, and (c) increase detection by engaging NK cellular co-receptors with ligands present on target cells. We used antibodies to the B cell protein CD20 and CD20-positive Raji B cells as a system to develop the assay. First, we defined conditions (e.g., duration time of assay) that maximized ADCC and minimized the NK activities that occur without antibodies. We then used the best assay time to titrate the antibody concentrations needed to trigger CD107a externalization using EC_50_s (the effective concentration of antibody needed to support 50% of maximal CD107a externalization). We compared EC_50_s for the WT and GE forms of GA101 that vary in fucosylation. Next, we pursued the unexpected finding that when NK cells were highly CD107a positive, there was little cytotoxicity towards the Raji cells. We proffer experimental calculations to reconcile this phenomenon. These assays were without brefeldin A or monensin because of the harmful effects of these reagents on viral protein expression [46,47,48,49]. These two reagents have been used to promote the detection of cytokine production by lymphocytes [24] and to limit CD107a endocytosis [19]. Last, we evaluated whether heat or formaldehyde treatment (used to reduce viral biohazards) would be acceptable.

### 3.2. Methodology for Detection of Externalized CD107a

**Figure A2:**
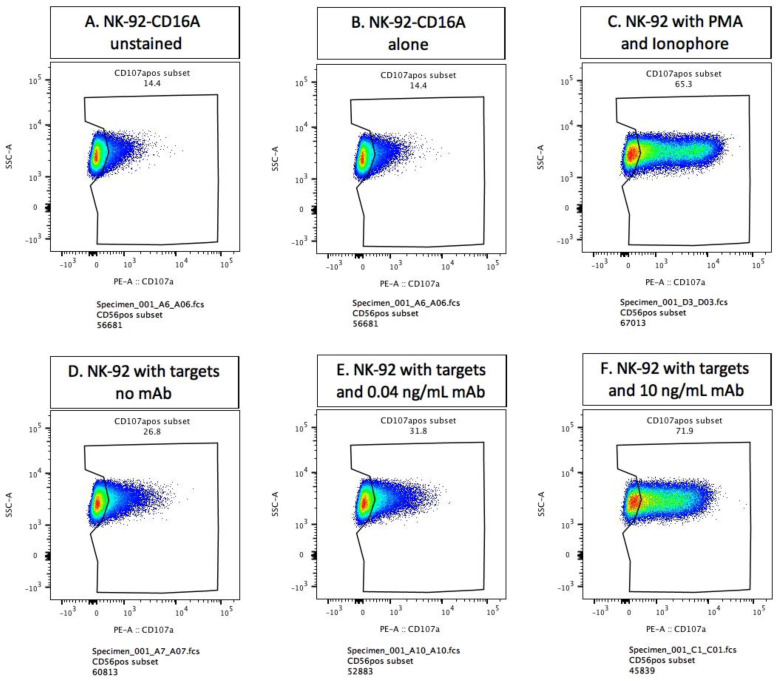
CD107a externalization after six assay conditions. The results are from JCA044 in which cells were cultured for 40 min, 1:2 NK to Raji target cells, without or with 0.04 or 10 ng/mL of GA-101 GE antibody or with phorbol myristic acid (PMA) and ionophore. (**A**) Unlabeled NK cells used to set gating for CD107a-positive cells. (**B**–**F**) Labeled cells. (**B**) NK-92-CD16A cells without Raji targets (repeat of Appendix A Figure A1F). (**C**) cells with PMA & calcium ionophore as a positive control. (**D**) NK activity towards Raji cells in the absence of anti-CD20 antibody. (**E**) NK plus Rajis with 0.04 ng/mL GA101 GE antibody. (**F**) NK plus Rajis with 10 ng/mL GA101-GE (repeat of Appendix A Figure A1E).

Raji cells were preincubated with dilutions of anti-CD20 and then co-cultured with NK-92-CD16A cells to induce CD107a externalization. Controls were NK-92-CD16A cells alone (for unstimulated expression of CD107a) and the NK cells plus Raji cells without antibody (for NK activity to the Raji tumor cells). After incubation, the cells were spun down and brought up to a minimal volume (~50 µL) for labeling with fluorescent antibodies. The volume was important, as the vendor’s PE-anti-CD107a was below saturation of CD107a; higher and/or uneven dilutions of the anti-CD107a affected signal detection. Appendix A Figure A1 and Figure A2 illustrate the gating of the GFP- and CD56-positive NK-92-CD16A cells to detect CD107a staining. We provide examples of unstained cells, stained NK-92-CD16A cells alone, PMA-ionophore stimulated positive controls, NK cells mediating natural cytotoxicity, and NK cells with low and high concentrations of anti-CD20. Of note, the percentage of CD107a positive cells determined by this manual gating was similar to that calculated by Overton subtraction of CD107a cells with Rajis without antibody from CD107a cells with Rajis and anti-CD20 antibody.

### 3.3. Quantification of Antibodies Required for CD16A-Dependent CD107a Externalization

NK-92-CD16A CD107a was better at detecting antibodies bound to Raji cells than fluorescent polyclonal donkey anti-human IgG (Figure 1). For these experiments, the effector NK to target Raji (E:T) ratios were 1:2 or 1:4, and the antibody concentrations varied. The EC_50_ for CD107a externalization in response to GA101-GE antibody was 0.22 ng/mL (1.5 × 10^−12^ M). This EC_50_ was ~7-fold more sensitive than the EC_50_ for flow cytometry of Raji cells with fluorescent polyclonal donkey anti-IgG to monitor bound anti-CD20 (Figure 1(A1)). The more sensitive detection by CD107a occurred despite the more detectable fluorescent intensities of the donkey anti-human IgG (Figure 1(A2)). Thus, CD107a externalization extends the limits of detection for antibodies that bind to cells.

We calculated upper limits for the number of antibody molecules per Raji that stimulated CD107a externalization. These limits could be calculated because we had information for the amount of antibody that was added to a fixed number of Raji cells and assumed complete binding as an upper limit. For the GE antibody, there was an average of 883 molecules that could be bound per Raji cell at the EC_50_ of 0.22 ng/mL. At the lower limit of *p* > 0.005 for CD107a detection with antibody, 0.16 ng/mL, there was an average of 627 molecules that could be bound per Raji cell.

NK-92-CD16A CD107a detected differences in antibody fucosylation. GA101 WT and GE are the same monoclonal antibody, differing only in post-translational glycosylation. Figure 1(B1) illustrates the percentage of NK cells that became CD107a-positive. The difference between the EC_50_s attributable to afucosylation was ~20-fold. Table 1 presents replicate experiments, highlighting in green the consistency of the EC_50_ differences attributable to afucosylation. An anomalous experiment JCA031 is included to show that differences in EC_50_s reflected afucosylation even when both EC_50_s in an experiment were aberrant. This anomaly emphasizes the need for an internal reference standard for the assay. In this case, the WT antibody serves as the reference standard.

The ~20-fold differences in EC_50_s are consistent with the antibody differences in afucosylation and the effects of afucosylation on antibody affinity for CD16A. There were 2.4-fold differences reported for the K_A_s for recombinant CD16A valine (produced in HEK-293T cells) with afucosylated vs. fucosylated IgG1 anti-CD20 [3]. There are five-fold differences in afucosylation between the WT and GE antibodies. Thus, CD107a EC_50_ differences are in line with affinity and afucosylation (2.4 × 5 = 12 fold). Additional effects are potentially attributable to the higher IgG1 affinity of CD16A from NK cells compared to the affinity of CD16A produced in HEK cells [50]. Antibody afucosylation also increased the MFIs of the cells with GE antibody (Figure 1(B2)), an observation consistent with more avid cellular CD16A binding to GE antibody.

### 3.4. Comparison of Antibody Concentrations Needed for CD107a Externalization and for Death by ADCC

It is possible that a single cytotoxic granule could be effective for killing while its associated CD107a might be below cytometric detection, which would mean that killing would require less antibody than CD107a externalization. However, the antibody EC_50_s for CD107a externalization and for target cell death (measured by ^51^Cr-release) were similar for both WT and GE variants of anti-CD20 (Figure 1(C1,C2)). Thus, the CD107a EC_50_s are good predictors of how much antibody is needed to support ADCC by the NK-92-CD16A cell line.

Comparison of the NK-92-CD16A cell line with peripheral blood NK cells is warranted to see if the NK-92-CD16A CD107a EC_50_s are likely to apply to cytotoxicity by ex vivo NK cells. NK-92 cells represent an early stage of NK cell maturation that has low cytotoxicity and a CD56^bright^CD16A^negative^ phenotype [51]. In contrast, >95% of NK cells in human peripheral blood are CD56^dim^CD16A^bright^ [52] and mediate high natural cytotoxicity [53]. Figure 1D indicates that the NK-92-CD16A CD107a expression correlates well with the antibody needed to support ADCC by peripheral blood NK cells. This correlation was applied to blood NK cells from donors with CD16A genotypes for AA158 phenylalanine homozygosity (F/F) as well as heterozygosity with valine (V/F). CD16A 158F has a 2-fold lower affinity for IgG1 than CD16A 158V [3] and 60–68% allele frequency in human populations [54]. Thus, the NK-92-CD16A cell CD107a externalization assay is likely to be relevant for predicting antibodies that will support ADCC by blood NK cells. Note that NK-92-CD16A cell lines may differ. Another laboratory, with NK-92-CD16A cells (obtained from Conkwest/NantKwest: San Diego, CA, USA), breast cancer target cells, and trastuzumab anti-HER2, found that blood NK cells were more effective for ADCC than their NK-92-CD16A cells [55].

### 3.5. Assay Conditions That Affected CD107a Expression

An optimal assay is short, simple, and easy to perform. Shorter times improve assay sensitivity because the background NK activity continues after ADCC stops. The NK cells lose their CD16A receptors during ADCC (cleavage by ADAM-17 [27]), while other NK receptors remain.

#### 3.5.1. Maximizing Detection of CD107a with Short Incubation Times

**Figure 2:**
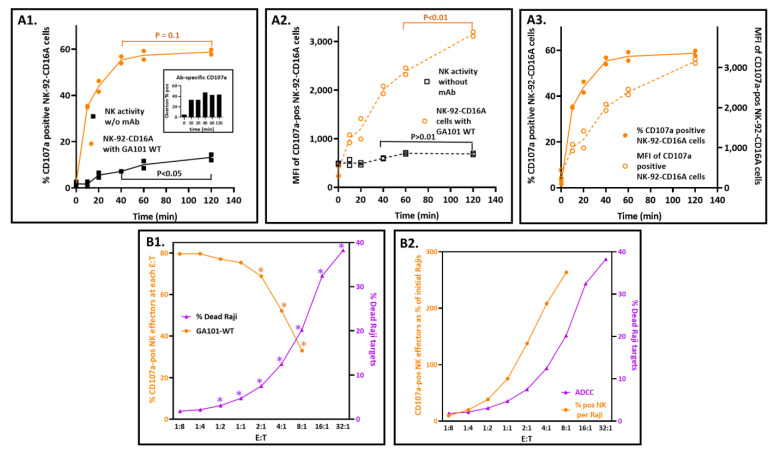
Conditions that affect CD107a externalization. (**A**) Time courses. The E:T was 1:2, and the antibody concentration was 1 µg/mL GA101 WT. (**A1**) Increases in the percentage of CD107a-positive NK-92-CD16A cells. The NK activity without antibodies is included to show its increase after antibody-dependent activity was complete. The inset illustrates that the antibody-dependent fraction (Total % − NK % positive) was unchanged after 40 min. (**A2**) Increases in CD107a expression. The MFIs are for the CD107a-positive cells from (**A1**). (**A3**) Side-by-side comparison of the % CD107a positive cells vs. CD107a. MFIs. The antibody-dependent data are from (**A1**,**A2**). (**B**) Effects of E:T ratios on CD107a externalization and death of Raji cells. The E:Ts varied from excess effectors to excess targets, as illustrated for two separate assays, one for NK CD107a externalization and another for ADCC by ^51^Cr release. Both assays were for 40 min with 1 µg/mL GA101 WT antibody. (**B1**) % CD107a-pos cells vs. death by ADCC. The percentage of cells with external CD107a paradoxically decreased with increased E:T ratios. Note: each datum for the % CD107a positive cells represents the % of a varying number of effector cells that increased two-fold for each E:T. The color-matched asterisks indicate *p* < 0.01compared to the E:T 1:8 values. (**B2**) Frequencies of CD107a-positive NK cell numbers vs. numbers of target cells killed by ADCC. The numbers (instead of percentages) of CD107a-positive cells at each E:T of (**B1**) were calculated and then re-expressed as percentages of the initial Raji cells, indicated in orange. The % Raji cell death is also from (**B1**). The CD107a-positive NK cells far exceeded the dead Raji cells (as indicated by the two ordinate scales). At the E:T of 8:1, the ratio of CD107a-pos NK cells to dead Raji cells was 13:1.

In these experiments, E:T ratios were 1:2, and CD107a externalization was arrested by EDTA to chelate the calcium needed for degranulation. The percentage of CD107a-positive cells reached near completion within 40 min for ADCC (Figure 2(A1)). Completion is indicated when CD107a externalization plateaued (at a maximum potential that is less than 100% of the NK-92 cell). In contrast, the CD107a associated with natural cytotoxicity continued. Comparing CD107a-positive populations with and without antibodies by Overton subtraction confirmed this interpretation (insert, Figure 2(A1)). Of note, the CD107a MFIs associated with antibodies continued to increase well beyond 40 min (Figure 2(A3)).

#### 3.5.2. Effector-to-Target Cell Ratios Had a Profound and Unanticipated Effect on CD107a Exocytosis

**Figure A3:**
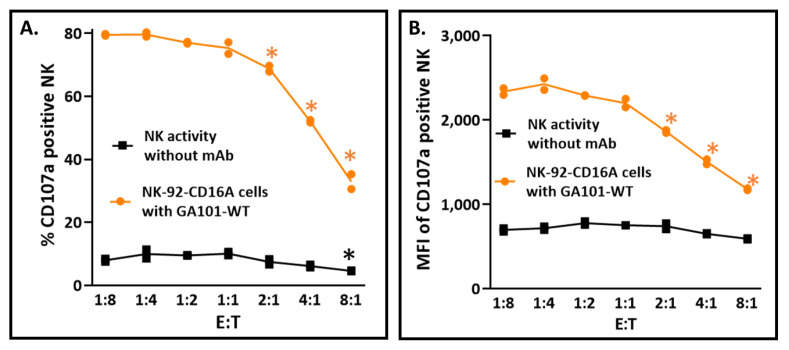
Comparison of CD107a-positive cells for anti-CD20 dependent and independent at increasing E:Ts. Assays without antibody (NK activity) and with antibody (supporting ADCC) were 40 min with 1 µg/mL GA101 WT antibody. * *p* = or <0.01 for *t*-test differences from the 1:8 E:T. (**A**) % CD107a positive cells. (**B**) CD107a MFIs. The MFIs are for the CD107a-positive cells of (**A**).

Since ^51^Cr-release was first used to measure cell death in 1968 [43], it has been observed that cytotoxicity increases as the ratio of NK or T effector cells to target cells (E:T) also increases. Thus, the finding that the percentage of CD107a-positive NK cells decreased as the E:T ratios increased (Figure 2(B1)) was unexpected. As expected, ADCC increased as a log function of the E:T. This inverse relationship also applied to natural cytotoxicity in the absence of antibodies (Appendix A Figure A3A). Furthermore, the CD107 MFIs also decreased proportionally with increased E:Ts, for both NK and ADCC activities (Appendix A Figure A3B). These findings are counter-intuitive to the expectation that antibody concentrations that induce CD107a externalization will simultaneously initiate ADCC!

The discord between the CD107a-positive NK cells and the targeted killing can be reconciled. The key to resolution is the recognition that the CD107a-positive cells are percentages of the effectors rather than fixed numbers of effector cells. The percentages were anchored in the two-fold increases in effectors at each E:T. When these percentages were converted into CD107a-positive NK cell numbers and compared to the numbers of dead Rajis, the relationship was concordant and positive. The CD107a-positive effector cell numbers were calculated by multiplying the percentages of CD107a-positive cells by the number of effectors present at each E:T (calculated in Appendix A Table A1). When the CD107a-positive NK numbers and Raji dead are both expressed as percentages of the starting Raji cells and graphed vs. the E:Ts (Figure 2(B2)), the slopes for CD107a and killing are both positive. The CD107a-positive NK cells greatly exceeded the dead Rajis at 40 min: at the E:T of 8:1, there were 264% CD107a-positive cells and 20.2% dead cells. The 13-fold ratio decreased to 2.6-fold at 2 h, consistent with rapid NK degranulation preceding slower target cell death. These ratios suggest that it takes multiple CD107a-positive effectors to kill one target cell. Thus, NK-92-CD16A cells are good for assessing antibodies that will engage CD16A and support CD107a externalization and poor for the prediction of actual target cell death.

### 3.6. Impact of Biosafety Treatments on the CD107a Assay

Heat inactivation and formaldehyde treatment are two means to abrogate viral infectivity. Heat inactivation of the serum is used to inactivate complement components (that could kill cells bound with antibodies) and is also used to inactivate viruses. Heat has the potential to denature the Fab and/or the Fc regions of antibodies. Potential effects include the formation of IgG aggregates that can bind to NK CD16A receptors and elicit CD107a externalization. Formaldehyde treatment is routinely used to abrogate viral infectivity. Treatment could be of virally infected cells after the addition of antibodies or later after the NK cells have reacted and prior to labeling them with PE-anti-CD107a. These treatments risk damage to proteins: damage to the Fc so that it no longer engages with CD16A or damage to the CD107a ligand so that the anti-CD107a antibody will no longer bind.

**Figure 3:**
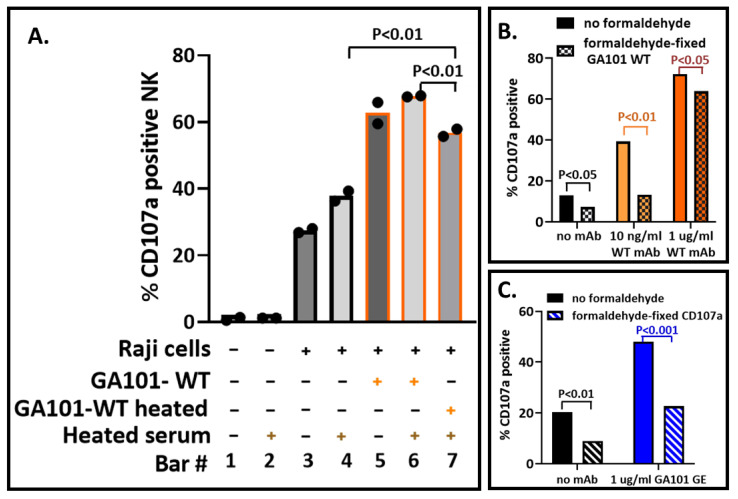
Variables that affect biosafety in future applications. Antibodies were heated to simulate inactivation of viruses. Formaldehyde treatment is routinely used to inactivate viruses. These assays were at an E:T of 1:2 for 40 min. (**A**) Heated antisera. GA101 WT antibody was heated with serum and then diluted with control heated serum to 1 μg/mL antibody in 10% heated human serum. The variables are indicated below each bar. (**B**) Formaldehyde fixation. Treatment was either (1) after addition of antibodies to Raji cells or (2) after the NK-92-CD16A-cells reacted with cell-bound antibodies. (**B**) Reactivity of the NK-92-CD16A cells to formaldehyde-treated GA101 IgG. Raji cells were treated with 1 or 0.01 µg/mL GA101 WT antibody, then 1% formaldehyde, washed, and used to stimulate NK-92-CD16A cells. The E:T was 1:2, and the assay was for 40 min. (**C**) Reactivity of PE-mAb anti-CD107a with formaldehyde-treated CD107a. Cells were treated with formaldehyde and washed prior to labeling.

Heat inactivation was at 56–60 °C for 30 min. GA101-WT mAb lost activity (bars 6 and 7, Figure 3A). However, some of its anti-CD20 activity was retained (bar 4 vs. bar 7). The data indicate that it would be optimal to avoid heat inactivation of antisera.

In the assay described, formaldehyde fixation was the last step prior to flow cytometry. Figure 3B illustrates that formaldehyde treatment at earlier steps is detrimental to the CD107a assay. The deleterious effects were greater at lower antibody concentrations and thereby reduced assay sensitivity. Formaldehyde treatment before labeling of the samples for flow cytometry indicated that CD107a lost antigenicity (Figure 3C). The binding of anti-CD56, anti-CD19, and anti-CD45 used to identify cells was unaffected, as was GFP fluorescence (not illustrated). These data indicate that formaldehyde treatment should best be used immediately prior to flow cytometry.

## 4. Discussion

We designed and optimized a cell-based bioassay to quantify antibodies that can support ADCC. The assay used a clonal anti-CD20 antibody with two levels of afucosylation as a proof of concept. We evaluated antibody concentrations, duration of the assay, and effector-to-target ratios to determine the conditions to support the most CD107a externalization by NK-92-CD16A cells. The assay detected as little as 0.2 ng/mL (1.5 × 10^−12^ M) antibody, was optimal at 40 min and at E:Ts with excess target cells, and was sensitive to antibody afucosylation. CD107a signals differed by ~20-fold in response to differences in antibody afucosylation. We observed a negative correlation between the CD107a externalization and target cell death mediated by ADCC and proposed a model below to address this conundrum. This assay, using immortalized NK-92-CD16A at E:Ts with excess targets, is a suitable basis for developing serum assays to characterize the ADCC potential of anti-viral antibodies to diverse viruses.

Details are important to optimize this assay. It is crucial to use a high concentration of fluorescent antibody to CD107a. GFP, translated after CD16A, helped identify healthy NK-92-CD16A cells. NK-92-CD16A lines developed by other investigators are likely to be comparably sensitive for use in CD107a assays [56] or even more sensitive (e.g., with CD16A as a fusion protein with domains of 41BB and CD3ζ to improve intracellular signaling [57]). Regardless of the cell source, it will be important to have E:T ratios with excess targets. The effects of E:T ratios on CD107a detection were profound. At the high E:Ts that supported ADCC cytolysis, there was marginal CD107a externalization. This phenomenon has been observed and reported before for NK cell killing without antibodies (see reference [25] Figure 4). In practice, for antibodies that can support ADCC, the E:Ts should be 1:1, 1:2, 1:4, or even lower. To assess anti-viral antibodies with infected cells, the assays will require careful attention to actual E:T ratios because only a fraction of the cells will be virally infected and able to become ADCC targets.

**Figure A4:**
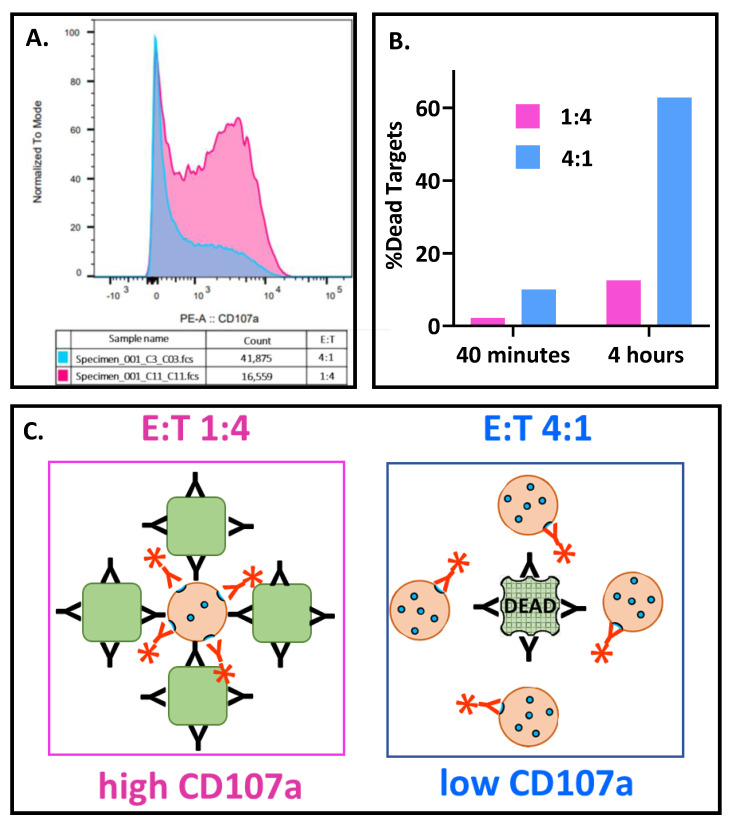
Model to explain why at the high E:Ts that support death, CD107a is hard to detect. CD107a externalization and 51Cr release were for 40 min with 1 µg/mL GA101 WT antibody. (**A**) Histograms of CD107a-positive cells E:T:Ts of 1:4 and 4:1 from Figure 2(B1). Note how many more CD107a-positive cells there are at the lower E:T of 1:4 and how much more CD107a the cells externalized. (**B**) Bar graph of ADCC at 1:4 and 4:1, from the data of Figure 2(B1) at 40 min and 4 h. (**C**) Model of additive attack to reconcile the low CD107a of the effector cells associated with killing. At low E:T, one effector releases multiple granules, but only one per target, which is insufficient to kill the target. At high E:T, each effector releases one granule and is barely CD107a-positive; however, the target received multiple hits and died. The black Y indicates the humanized anti-CD20 mAb that directed ADCC; the orange Y indicates the mouse PE fluor (*)-tagged mAb anti-CD107a.

The unexpected disconnect between low CD107a and high ADCC has ramifications for the interpretation of CD107a externalization in tumor and virally infected cell microenvironments. *How can optimal killing occur with a low release of cytotoxic granules*? We advance a model to explain this phenomenon (Appendix A Figure A4). At a low E:T (e.g., 1:4), one killer cell may attack multiple target cells without killing any of the targets. We postulate that the effectors released too few granules *per target* to be lethal. The high CD107a of each outnumbered killer cell indicates that the killer spent a lot of granule ammunition futilely, probably engaging in multiple sublethal attacks. At a high E:T (e.g., the inverse 4:1 ratio), multiple NK cells can attack a single target cell simultaneously. Together, the NKs deliver sufficient ammunition to kill this target cell, and then they may halt their cytotoxic granule release after the target dies [58]. The low CD107a of each of these identified killer cells indicates each one released only a small amount of its stored granule ammunition. NK-92 cells can kill with as few as three granule externalizations (detectable with a CD107a-GFP construct and confocal microscopy [59]), which suggests that the threshold for detection of degranulation by flow cytometry may be too high to detect all the killers at high E:T ratios. Nonetheless, multiple killers, probably working together, spent sufficient granule ammunition to cause death while outnumbered killers failed. One ramification of our model is that low or undetectable NK or T cell extracellular CD107a in vivo may actually be associated with cytotoxic activity!

We contrast our assay with assays developed by other investigators. We focus on assays designed to detect differences in specific antibody afucosylation without the use of mass spectrophotometry. For the assay presented, the effects of afucosylation would be relative to an IVIG or a mAb standard for specific anti-viral antibodies. We compare three alternative assays by these criteria: sensitivity, simulation of viral antigens, the structure of CD16A, laboratory time, and quantitation. Simulation of physiological antigens is important since infected cells will display multiple viral proteins. NK cell CD16A binds better to IgG than CD16A produced in HEK cells [50]. Quantitation of the amounts of anti-viral antibodies provides insight into molar antibody concentrations and how far they might be able to drop before losing bioactivity.

One assay utilized infected cells as the source of viral antigens and a T cell tumor line transfected with CD16A as the sensor for anti-viral antibodies [60]. After CD16A recognition of cell-bound anti-viral antibodies, the T cells secreted IL-2, which was measured by ELISA. This design permitted the detection of antibodies specific to many viruses that were able to support ADCC. This assay was sensitive to 640 ng/mL monoclonal anti-RSV IgG1 and had the advantage of viral antigens in physiological protein conformation and at infectious densities of proteins in the plasma membranes. It is a ‘universal’ assay and will detect antibodies to any virus that generates antigens in infected cells’ plasma membranes. A disadvantage of this assay is the time needed for the T cells to produce IL-2 and for the IL-2 ELISA.

Two other assays that detected afucosylated anti-viral antibodies utilized recombinant (r-) viral protein antigens, either bound to enzyme-linked immunosorbent assay (ELISA) plates [61] or to beads for flow cytometry [62]. These assays relied on r-CD16A protein for antibody detection (rather than cellular CD16A). The assay called Fucose-sensitive Enzyme-linked ImmunoSorbent Assay (FEASI) [61] used SARS-CoV-2 r-spike (S) protein as the antigen, with two ELISA read-outs: (1) for anti-human IgG for antibody quantification and (2) for biotinylated monomeric r-CD16A followed by enzyme-linked avidin. A mAb IgG1 anti-S protein with varying percentages of afucosylation was used for the calibration of afucosylation. The assay provided an excellent assessment of afucosylation but required 100 ng/mL specific mAb when the antibody was 4% afucosylated. This assay is time-efficient because it is independent of tissue culture, quantitative, and is suitable when specific antibodies are elevated in serum. Its disadvantages are limited sensitivity and restriction to a single r-viral protein.

The flow cytometric ‘Fc-array’ assay [62,63,64] employed color-coded beads bearing diverse r-viral antigenic proteins combined with different Fc-receptors that included CD16A. HIV and influenza proteins were coupled to the beads. Specific antibodies bound to the beads were detected with (a) PE-tagged antibodies to IgG subclasses and (b) PE-tagged avidin molecules containing four biotinylated r-CD16As per avidin. The CD16A tetramers could detect 1 × 10^−9^ M mAb anti-HIV [63] with less sensitivity than the NK-92-CD16A assay. PE-tagged, fucose-sensitive lectins were also used as probes. The Fc-array is suitable for calibration with an anti-viral mAb with different levels of fucosylation and for quantification of the bound anti-IgG1 and IgG3. Its current disadvantages are dependence on ratios of qualitative MFIs for bead-bound anti-IgG1 and r-CD16A. The r-CD16A produced in HEK cells may have reduced sensitivity compared to NK cell native CD16A.

The ‘NK-CD107’ assay described here has advantages inherent in its biology. It does not require high-affinity antibodies. It can detect less than 600 molecules of antibody bound per target cell, well within levels of cellular viral protein expression. The assay will work with many different viruses, even lytic viruses that have killed the host cells but left viral antigens behind in the dead cell membranes. It can be rapidly produced to detect emerging infectious viruses: all that is needed is one human source of antibodies and one positive control viral stock for application to detect infectious viruses in unknown samples.

The ‘NK-CD107’ assay will need further development in order to evaluate anti-viral antibodies that will support ADCC. To quantify anti-viral antibodies rather than titer them, one could measure the IgG 1&3 bound to infected cells by flow cytometry. An accepted method [63] utilizes phycoerthrin (PE)-labeled monoclonal anti-human IgGs 1&3 (with one PE molecule per mAb molecule) and PE standards [65] to quantify the bound antibody by flow cytometry. We recommend that the infected target cells be human or primate in origin. The species compatibility may be necessary for effector NK cell receptor to bind to target cell counter-ligands, e.g., the interaction of NK CD2 with target cell CD58 that promotes lysis [66].

The viral inocula used for infections and the post-infection time of expression of plasma membrane viral proteins will require careful attention. An early example of ADCC to the coronavirus (229E) indicated feasibility for the detection of naturally infected cells and that timing was important for cell surface expression of sufficient antigens [67]. A reference anti-viral antibody standard, such as a humanized anti-viral mAb or an intravenous immunoglobulin preparation IVIG [63], will be needed to control for inter-assay variation. The advantages of this approach are viral proteins with native protein structures in physiologically relevant concentrations and the ability to detect multiple viral proteins. For example, the coronavirus OC-43 has a hemagglutinin as well as a spike protein that will be found in plasma membranes. Intact viruses, with their multiple envelope proteins, may also be displayed for ADCC when the viruses are bound to infected cells by tetherin [68,69].

We have characterized a sensitive cell-to-cell-based assay to detect antibodies that support ADCC that also has unique potential for physiological insights.

## 5. Conclusions

In summary, we have advanced assays for anti-viral antibodies that support ADCC. The NK-CD107a assay increases the sensitivity of detection and encompasses antibody afucosylation. It is ‘universally’ adaptable to different viruses.

## Figures and Tables

**Table 1:** EC_50_ comparisons for low and high afucosylated antibodies for externalization of CD107a *.

Exp. No.	EC_50_ (ng/mL)			E:T NK:Raji	% CD107a Positive Cells ^a^			Median Flourescent Intensities (MFIs)		
	10% aFucosylated GA101 WT	50% aFucosylated GA101 GE	Ratio WT/GE		ADCC w/Highest ab conc	NK w/o ab	ADCC Minus NK	ADCC at Highest ab conc	NK w/o ab	ADCC-Minus NK
JCA044	4.6	0.23	20	1:2	75.3	35.4	39.9	1034	495	540
JCA042 & JCA043	5.0		24	1:2	64.1	19.4	44.7	1795	1257	538
		0.21			47.8	4.6	43.2	1166	521	646
JCA036	3.2	0.09	35	1:4	79.0	21	58.0	1396	892	504
JCA039	ND	0.25	NA	1:2	89.5	36.5	53.3	3080	908	2172
JCA049	1.4	ND	NA	1:2	85.9	52.15	33.8	4129	1846	2283
**Averages**	**3.5 #**	**0.2 #**	**26**		**73.6**	**28.2**	**45.5**	**2100**	**986**	**1114**
**St dev**	**1.6**	**0.1**	**7.4**		**15.4**	**16.6**	**8.9**	**1239**	**507**	**865**
JCA031 **	66.7	2.9	23	1:4	51.8	5.7	46.1	1563	962	601

^a^ The %CD107a-pos cells of CD56posGFPos NK-92 cells. # Differences between the 10% and 50% fucosylated antibodies, *p* < 0.01. * Assayed at 40 minutes. Green background indicates the differences due to fucosylation of the antibodies. Bold font indicates the averages and standard deviations of experiments with similar EC_50_s. ** This experiment had a WT/GE EC50 antibody ratio in common with the other experiments but both EC_50_s were inexplicably high.

## Data Availability

All related data and methods are presented in this paper. Additional inquiries should be addressed to the corresponding author.

## Appendix A

**Table A1:** Method for calculation of normalized data, Figure 2(B2,B3) *.

Data from JCA054						Data JCA055	2B4 Calculated CD107a pos NK per Dead Target at 40 min
Expt’l Conditions, Figure 2			2B1 Primary Data, NK w/Raji & ab; % CD107a pos NK	Calc as Number CD107a-pos NK Cells per 10,000 Targets	2B3 & 2B4 Normalized % NK CD107a pos per Initial Raji Target	2B3 Primary Data % Dead of Initial Raji Cells	
E:T	NK Cells *	Raji Targets *					
**0.125 to 1**	**1250**	**10,000**	**79.55**	**994**	**9.9**	**1.84**	**5.4**
**0.25 to 1**	**2500**	**10,000**	**79.65**	**1991**	**19.9**	**2.16**	**9.2**
**0.5 to 1**	**5000**	**10,000**	**77.05**	**3853**	**38.5**	**3.1**	**12.4**
**1 to 1**	**10,000**	**10,000**	**75.35**	**7535**	**75.35**	**4.74**	**15.9**
**2 to 1**	**20,000**	**10,000**	**68.8**	**13,760**	**137.6**	**7.52**	**18.3**
**4 to 1**	**40,000**	**10,000**	**52.1**	**20,840**	**208.4**	**12.51**	**16.7**
**8 to 1**	**80,000**	**10,000**	**32.95**	**26,360**	**263.6**	**20.21**	**13.0**
**columns**	**B**	**C**	**D**	**E = B × D**	**F = E/100**	**G**	**=F/G**

* Colors in table are the same as for the symbols in Figure 2. The experimental conditions are normalized to 10,000 Raji target cells for illustration, but were 250,000 Raji per tube. NK cell numbers are based on the E:Ts. The calculations are indicated by letters below each column. Both the CD107a assessment and the 51Cr dead was determined at 40 minutes.
